# A reflection on HIV/AIDS research after 25 years

**DOI:** 10.1186/1742-4690-3-72

**Published:** 2006-10-20

**Authors:** Robert C Gallo

**Affiliations:** 1 Institute of Human Virology, University of Maryland Biotechnology Institute and Department of Microbiology and Immunology, University of Maryland School of Medicine, Baltimore, Maryland 21201, USA

## Abstract

Dr. Robert C. Gallo provides a personal reflection on the 25 year history of AIDS.

## Reflection

**Figure 1:**
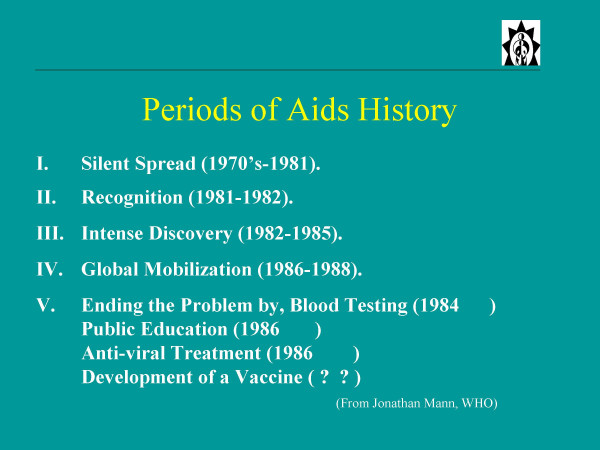
A summary of the five periods of AIDS history as modified after Jonathan Mann.

A reflection on the 25 year history of AIDS can begin with no better outline than that provided by the late Jonathan Mann of WHO. A slide he gave to me in the late 1980's divides the history of AIDS into four periods: (see fig. 1). Jonathan could not know that the period of silent spread (part 1 of this saga) of HIV actually began years earlier. We now know that, by 1971, the virus had moved to several different regions of the world, but exactly when it came out of Africa is conjectural.

There has been considerable attention (no less than three papers in *Science *and *Nature *over the past few years from B. Hahn and her colleagues) that the natural reservoir for HIV-1 is a particular subspecies of chimp [1-3]. The primate-to-man origin of HIV was suspected almost from the beginning, albeit without knowing which primate. The reasons were three fold: 1) the early evidence that HIV was widespread in central Africa; 2) the evidence that HIV was more variable in Africa (hence longer presence); and 3) prior experience with HTLV-1 and HTLV-2 and their related retroviruses in African and Asian primates (STLV strains), especially the evidence suggesting a chimp origin of HTLV-1, coupled with the discovery of SIV by scientists in Boston, and later of many other strains identified in various African but not Asian monkeys. Human sera reacted better with some of these SIV strains from West Africa than they did with HIV-1, giving impetus to the work that led to the finding of HIV-2, and the obvious conclusion that HIV-2 came into man from these monkeys (sooty mangabey) [4].

But, how did the original infection of people in rainforests become an epidemic? Here we must rely on history. I presume people in rainforests (especially hunters) were occasionally infected for a long time, but died with their disease. Migration to cities may have been associated with increased prostitution. The movement of the rainforests to the world can be seen as the consequence of post World War II societal changes: increased travel with increased promiscuity, advancing intravenous drug addiction, and blood and blood products moving from one nation to another for medical purposes.

Part 2 (Fig. 1) is the identification of the disease by U.S. clinicians (1981) [5-7], and defining it as an immune disorder characterized by a decline of immune function and of T cells, and notably CD4 T cells, and by 1982 the identification of risk groups then called the "4 H's" (hemophiliacs, heroin addicts, homosexuals and Haitians). It is in the period (1982) when my colleagues and I began to think about this problem, and we initiated our first experiments in May 1982.

Along with Max Essex in Boston, I speculated in early 1982 that AIDS would be caused by a retrovirus. This was based on information that some retroviruses, like feline leukemia virus (FeLV), caused not only leukemia, but blood cell deficiencies including those of T cells [8]. This was apparently associated with genetic changes in the FeLV envelope. More importantly, I was influenced by our experiences with human retroviruses (HTLV-1 and HTLV-2), which we had only recently discovered [9-11]. The reasons were six fold: 1) HTLV-1 and HTLV-2 mainly targeted CD4 T cells; 2) we knew they were transmitted by blood, sex, and mother to infant especially by breast feeding. These were precisely the suggested modes of transmission of the putative microbial cause of AIDS suggested by James Curran of the U.S. Centers for Disease Control (CDC); 3) HTLV's were endemic in parts of Africa and in Haiti, and CDC had announced these were hot-beds for AIDS; 4) we knew that, even in the absence of leukemia, HTLV-1 could cause minor immune impairment; 5) we had just discovered HTLV-1 and HTLV-2, so why not a 3^rd ^human retrovirus, and one with the capacity to cause a profound immune disorder? 6) finally, as we began this work somewhat tentatively, I was encouraged by David Baltimore, who independently wondered aloud to me that a retrovirus was probably the origin of AIDS.

The idea, however, has sometimes been misunderstood and misrepresented as our hypothesizing that HTLV-1 itself was the cause of AIDS. That is clearly not the case. Our idea was that AIDS would be caused by a new retrovirus, but one in the HTLV family. At the time, there were at least a dozen theories as to the cause of AIDS, including non-infectious causes. Our hypothesis was the one that bore fruit. As we soon learned to our astonishment, HIV would be in a separate family of retroviruses.

J. Mann's Part 3 of AIDS history are the years 1983–85. He called this the period of intense discovery. It begins with the isolation of HIV. Our approach to find the virus of AIDS was to follow our successful pattern with the HTLV's, namely, the culture of blood cells from patients, activation of the T cells in these samples, growth of the T cells with IL-2, and search for reverse transcriptase actively in the supernatant. If positive, we would look for some cross reactivity with HTLV-1 or HTLV-2 with antibodies to proteins of these viruses. Concomitantly, we probed DNA and RNA of some primary tissues of AIDS patients using cDNA from HTLV-1 under rather relaxed conditions in order to detect sequences that might be related to HTLV-1 and 2. In 1982 and in early 1983, these experiments gave variable results that were sometimes highly positive, other times borderline or even negative. In retrospect, the highly positive samples (with sequences related to HTLV) were due to patients being doubly infected with HTLV-1 or HTLV-2 plus HIV, which occurred in close to 10% of our samples. Negative or borderline RT positive samples were due to our performing the RT assays later than the optimal peak of virus production, which occurs days earlier with HIV than with HTLV. Luc Montagnier was stimulated in part by our ideas brought to France by the French clinician Jacques Leibowitch and, in early 1983, I sent Montagnier IL-2 and antibodies against the HTLV's. He and his co-workers had found evidence of a retrovirus in a patient with lymphadenopathy, and they could distinguish it from the HTLV-1 and with those antibodies [12]. This was the first "clean" finding of HIV. Our samples at that time always had HTLV-1 as the dominant virus. However, by mid 1983, we were able to obtain many isolates of HIV, and by the time we published our papers (May 4, 1984) we described isolates from 48 patients [13]. Importantly, we were able to put six of these isolates into continuously growing T cell lines [14].

This was the necessary breakthrough, because for the first time there would be sufficient virus for detailed characterization and the development of a workable HIV blood test. The blood test (for serum antibodies to HIV), along with the large number of isolates from AIDS patients, were the major convincing results that HIV (which at the time we called HTLV-III and the French group called LAV) was the causative agent of AIDS [15].

Demonstrating that HIV was the cause of AIDS provided some special challenges – unlike most viral infections. The first was the long period between infection and the signs of AIDS (5 to 15 years). Physicians and public health officials do not ask a patient what they did a decade earlier, but rather think in terms of days or weeks. The second was the numerous infections a patient develops as they present with AIDS. Which one, if any, was the cause? The third was our concerns about verification. For rapid progress, it was essential to have rapid verification, and there were at least two factors that could greatly prolong achieving this goal. (1) Samples from AIDS patients were not only limited, but some institutions had forbade even their entry due to fears of infection. (2) T-cell culture technology, though available in immunology laboratories, was not widely available in virology laboratories. Both of these restrictions made it unlikely that there would be sufficiently rapid and conclusive confirmation by HIV isolation. Consequently, the blood test seemed to us to be particularly urgent for three reasons: 1) it allowed prevention of HIV transmission from contaminated blood; 2) it opened the door to our ability to follow the epidemic from the early period of infection, and 3) it provided for verification of HIV's causative role in AIDS. The test for serum antibodies was simple, inexpensive, safe, rapid, sensitive and accurate. Consequently, verification came rapidly and globally.

A problem then occurred that enormously hindered our work over the coming years. One of our culture samples became contaminated with virus sent to us by Luc Montagnier. At first we stubbornly refused to believe that this was possible, because the strain of HIV from Paris had different characteristics in cell culture. However, this has now been clarified [16,17]. Montagnier had unknowingly sent us a very different strain of HIV that grows well in cell lines. This strain contaminated his culture of LAV before it contaminated one of ours.

Then, from all sides and in big doses, came patent suits over royalties to the blood test, lawyers, media, politics and just plain pressure. Meanwhile, there were other odd problems such as people who denied the existence of AIDS, others who believed HIV did not exist, groups who believed HIV existed, but didn't cause AIDS, and those who believed HIV existed, caused AIDS, and was developed in a U.S. laboratory to kill African Americans and gay men. Suffice it to say, no scientist is prepared for things like this. Despite these distractions, science progressed with great speed. Mann called it the fastest pace of discovery in medical history from the time of inception of a new disease.

To briefly revisit that period, some of the noteworthy advances are listed here. They include discovery of HIV (1983–84) [12-14]; convincing evidence that it was the cause of AIDS ('84) [15,18,19]; modes of transmission understood ('84–'85); genome sequenced ('85) [20-22]; most genes and proteins defined ('84–'85) though not all their functions[23-25]; main target cells CD4 T cells, macrophages, and brain microglial cells – elucidated [26,27]; key reagents produced and made available for involved scientists all over the world ('84–'85); genomic heterogeneity of HIV ('84) – including the innumerable microvariants within a single patient ('86–'88) [28,29], first practical life saving advance ('85); the blood test ('84)[30]; close monitoring of the epidemic for the first time, because of the wide availability of the blood test ('85); the SIV-monkey model ('85) [31,32]; the beginning of therapy – AZT ('85)[33]; and the beginning understanding of pathogenesis ('85)[34].

These rapid advances led to expectations that AIDS might be quickly solved. However, those scientists with experience in retrovirology knew differently: Unless a successful vaccine was soon available, this would be a long road – an infection that might be permanent in the population as retroviruses are in many species. Furthermore, we knew by mid-1984 that the infection was becoming global. We had tested sera from many countries, and we could follow the evidence for HIV coming into a region (a positive HIV blood test) with subsequent AIDS. However, we could never anticipate the HIV African tragedy.

Despite the rapid advances in those years, I think it is still appropriate to ask whether we could have done better. For example, were we as medical scientists, health officials, doctors or simply as members of society prepared? The answer is an interesting mix of opposites! On the one hand, if AIDS had to come, we were lucky that (scientifically speaking) it came at a very good time. The 1970's saw the revelation of the replication cycle of animal retroviruses (so we had a framework to work by once HIV was established as the cause). We had most modern tools of molecular biology (mainly developed in the 1970's). We had monoclonal antibodies also developed in the 1970's. We had access to technology to grow human T-cells with IL-2 which my colleagues and I developed in the mid-1970's, and we had found other human retroviruses in the 1980's-82 giving the first credence to their presence in humans. However, if AIDS had to come, we could also say it came at the worst of times. It seems that people have a memory span not longer than 25–30 years. Here are three examples of what I mean: First, was the surprise and lack of preparation in 1918–1919 for the great influenza epidemic – forgetting lessons of the late 19^th ^century [35]. Secondly, there was surprise and lack of preparation at the onset of the polio epidemic in the late 1940's and early 1950's [36]. It is eerie to read accounts of that period showing that medical science in particular and society as a whole, were focused on chronic degenerative diseases, believing serious infectious diseases to be "conquered". Eerie also because, thirdly that was precisely the attitude once again by the late 1970's evidenced by the closure of some microbiology departments, and threats of increasing reductions to CDC. The microbe would be simply the playground of the molecular biologist. Some even felt humans could not be infected by retroviruses.

No group was really responsible for unraveling the cause of the new epidemic, except the CDC, but in my view the CDC cannot and does not have expertise in every class of microbes, let alone for all types of viruses, and indeed they had no expertise in animal or human retroviruses. Our group became involved only after I listened to a lecture by the CDC's James Curran, who called for help from virologists. I have suggested that the government provide base support for 10 or more virus centers, covering all types of viruses among the centers. These centers would be responsible for providing needed expertise to the CDC for the etiological agent, diagnostics and possibly therapy and prevention. In accordance with the kind of virus suspected, the center(s) would be activated. Each center might also be required to have close collaborations with at least two groups from developing nations.

Though the HIV blood test was brought forward rapidly (early 1985) to large companies that could make the test available on an industrialized scale, I believe we could have still done better. For instance, we could have tested the pooled plasma used for hemophiliacs in 1984 without a large industrial scale production of the test. I don't think anyone was thinking of this. We were advised to return to basic laboratory research and assumed someone would be doing these tests. The lesson here for me is to take more control of things that come from your own work.

Where did things go since this early period of 1982–85? Jonathan Mann describes Part 4 ('86) as the time of global mobilization. This means education leading to prevention of infection, and no doubt this was the second major practical advance and it continues today with results that vary in place and even in time. There is proven success in some places, but not all, and sometimes there is only temporary success. It is noteworthy that appropriate education also depends upon the blood test, hence on basic science.

There were many other major advances over these next 20 years ('86-06), but none were more important than therapy. This is listed as Part 5 of Mann's summary, but it was added by me as the "period" era of practical advances, but the time lines for these advances are actually from 1984–1995. AZT showed for the first time that a viral disease could be objectively treated (decline in virus levels and lessened signs of AIDS), and there is no need to embellish here on the great advances made with the triple drug therapy in the mid 1990's. This was from contributions of a great number of scientists: those who contributed to our basic understanding of HIV replication, and as a result to targets for therapy, and those who developed the culture systems to grow HIV that could also be used in drug testing, and of course to the pharmaceutical industry, especially those like E. Emini who helped design and develop the protease inhibitors.

The other major practical advances in the last two decades have mainly been an extension of the earlier ones: more widespread use of the blood test as well as educational programs; refining therapies; learning about HIV drug resistance and how best to avoid it; better care of patients; and learning about serious co-infections especially of tuberculosis and HCV. A selection of the most important basic science advances will be debatable. In my view, the most important include the following: clarification of the two HIV strain functional extremes – the pure CCR5 tropic viruses and the CXCR4 tropic viruses [37-42], for review, see [43,44]; the discovery of the first endogenous inhibitors of HIV (β-chemokines) [44]; elucidation of the mechanisms involved in the action of some the HIV non-structural proteins [45,46]; an appreciation of the role of abnormal immune activation in pathogenesis, which impacts not only HIV infected cells, but is also detrimental to uninfected immune cells for review, see [44,47]; major advances in our understanding of the various types of HIV in different regions of the world and new recombinant forms; evolving knowledge of the envelope structure [48,49]; the details of HIV entry into cells [50]; and various genetic and some environmental mechanisms for resisting infection and slowing progression to AIDS in infected persons, as well others fostering infection and progression [51,52]. These latter basic advances have already had their practical impact, including for example several new approaches for drugs that target HIV entry.

We have reached the end of the first 25 years of AIDS, and we can safely say that we know as much about HIV as we do of any pathogen and about AIDS as we do any human disease. The remaining problems and needs are evident: bringing therapy and better health infrastructure to poor nations; continuing to develop new treatments because of the need for life-long therapy and the associated drug side effects and HIV resistance; continuing and advancing education; global monitoring of the different strains of HIV for changes in their virulence, transmissibility and drug resistance; and development of a preventive vaccine which provides sterilizing immunity (or close to it) [53]. For a successful vaccine, I believe we need neutralizing antibodies that are sustained (do not need rapid recall), and I think this is a reachable objective. Parenthetically, it has often been erroneously said (most recently in the *New York Times *reporting of the Toronto International AIDS Conference) that, at the April 1984 press conference, Secretary of Health, Margaret Heckler stated that a successful vaccine would be available within two years. Transcripts of comments are still available and show that no such claim was made. Rather, it was said that the virus could be continuously produced in large amounts, thereby making trials possible within two years. Indeed, this proved true, because in 1986, Daniel Zagury carried out some phase 1 trials in Africa and in Paris.

Several encouraging developments provide some optimism for the future, such as the ability of some nations to diminish rates of infection by education, and major new funding sources aimed at practical achievements. One laudable example is President Bush's Emergency Plan for AIDS Relief, which is providing $15 billion for therapy for HIV positive patients in needy nations. We have been impressed that this effort carries out its mission with leadership by university clinical scientists who work with groups with long experience in the specific country. In contrast to alternative plans that simply and rapidly provide funds for the drugs, these programs add to local infrastructure and training, thereby reducing the prospects for creating more multi-drug resistant HIV mutants. Private foundations have also been a new forceful addition; International AIDS Vaccine Initiative (IAVI) for developing vaccine candidates and the Bill and Melinda Gates Foundation for fulfilling many needs.

There are also major concerns for the future. We know science is essential for solving the HIV problem and, as noted before, science has been responsible for all the major practical advances in fighting this disease. However, there is a growing distance between scientists and the larger public. John Moore of Cornell Weill Medical School reminded me that C.P. Snow wrote about this in the 1950's, but I think the gap has continued to widen as technology becomes more and more specialized. Sometimes, it leads to tension and even hostility by the larger public toward scientists. This is sometimes evident in AIDS, seemingly so in recent years. Consider a recent CNN program that was a positive educational force, but advertised as one composed of AIDS experts. However, not one scientist was among the experts, and the program ended with a movie actor stating that to solve the problem, we all "had to be together." It was togetherness rather than science that he informed us would solve AIDS.

To return to and end on a positive note: it is interesting and useful to remember that there has been some silver lining on the dark AIDS clouds. Consider the many positive spin-offs to science in immunology, cancer biology, basic virology, and even molecular biology along with the leadership and focus AIDS research has provided to therapy of viral infections and to vaccine development. Positive spin-offs have not been limited to science. Consider how AIDS has inspired far greater tolerance (at least in the West) of differences in sexuality and much greater scientific and humanitarian collaborations between developed and less developed nations. Certainly, this is the case for relations between the U.S. and Africa. Let us hope these advances in understanding and in conscience will continue to evolve and grow so that there will be no need for anyone to reflect on AIDS in its 50^th ^birthday year.
